# Supplementary material for: Correlation of Electrophysiological and Fluorescence-Based Measurements of Modulator Efficacy in Nasal Epithelial Cultures Derived from People with Cystic Fibrosis
Source: Cells. 2023 Apr 17;12(8):1174. doi: 10.3390/cells12081174 (PMC10136647; doi:10.3390/cells12081174)
Supplement: Supplementary file 1 [file cells-12-01174-s001.zip › cells-2291722-supplementary.pdf]

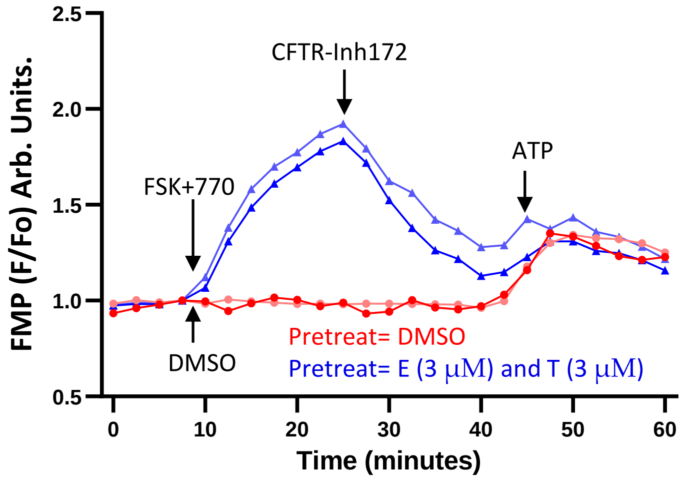

Figure S1: FMP traces of primary nasal epithelial cells studied in SpectraMax i3X plate reader. Primary nasal cells from a donor with the genotype (F508del/I507del) were cultured for 14 days under ALI conditions in 24 transwell plates and treated with DMSO or Elexacaftor (E) or Tezacaftor (T) for 48 hrs. Traces show the duplicate responses to acute treatment with agonists: FSK (10 μM) and VX-770 (1 μM) , CFTRinh-172 (10 μM) and ATP (100 μM).

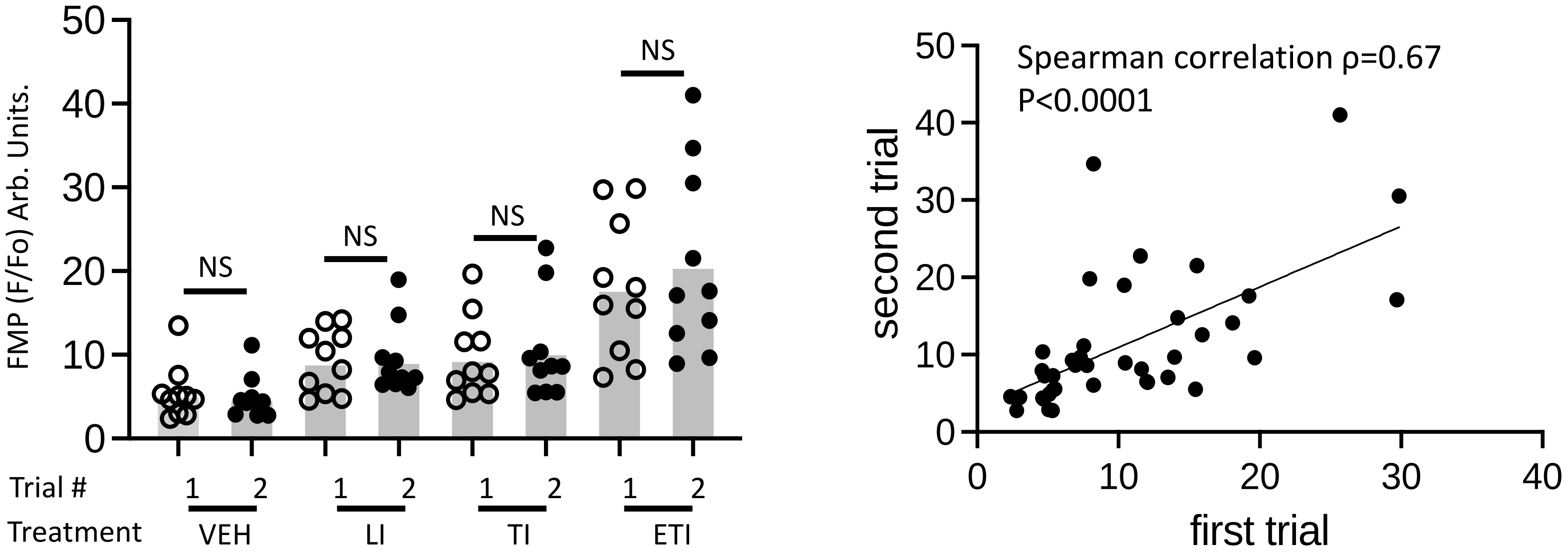

Figure S2: Scattergram shows that there are no differences in the in vitro (stimulation) responses measured for cultures generated from two different frozen vials for 10 donors (genotype=F508del/F508del). Responses were measured after pretreatment with VEH (DMSO), Lumacaftor (L), Tezacaftor (T) or Elexacaftor plus Tezacaftor (ET). Acute stimulation was induced by forskolin plus ivacaftor (I) as in Figure 1 and 2. The graph to the right shows the correlation between Trial #1 and Trial #2 for responses to modulators (or DMSO) for the 10 donors.
